# Orbital lymphoma: diagnostic approach and treatment outcome

**DOI:** 10.1186/1477-7819-11-73

**Published:** 2013-03-18

**Authors:** André M Eckardt, Juliana Lemound, Majeed Rana, Nils-Claudius Gellrich

**Affiliations:** 1 Department of Cranio-Maxillofacial Surgery, Hannover Medical School, Carl-Neuberg-Strasse 1, Hannover, 30625, Germany

**Keywords:** Orbital lymphoma, Mucosa-associated lymphoid tissue lymphoma, Radiotherapy

## Abstract

**Background:**

Lymphomas of the orbit and orbital adnexae are rare tumors, comprising only 1% of all non-Hodgkin’s lymphoma. The majority of non-Hodgkin’s lymphomas of the orbit are extranodal marginal-zone B-cell lymphomas of mucosa-associated lymphoid tissue type. Because of nonspecific clinical signs and symptoms, some diagnostic delay may occur. The purpose of the study was to evaluate the diagnostic approach in orbital lymphomas and to analyze their treatment outcome.

**Methods:**

In the period from 2005 to 2012, from a group of 135 patients with tumors of the orbit, we identified 11 patients diagnosed with orbital lymphoma. This patient cohort was reviewed retrospectively.

**Results:**

The patient group consisted of 11 patients (seven females, male males) with a median age of 57.7 years (range 42 to 88 years). Orbital swelling, pain and motility impairment were the leading clinical symptoms. Diagnosis was confirmed by surgical biopsy. Depending on the anatomic location of the tumor, a surgical biopsy was taken using a blepharoplasty incision, a lateral orbitotomy or a navigation-guided biopsy. The predominant histology was extranodal non-Hodgkin’s lymphoma of mucosa-associated lymphoid tissue type (82%). All patients underwent complete clinical staging. These were clinical stage I_EA_ in seven patients, and stages II_EA_ (*n* = 2) and III_EA_ (*n* = 2) in four patients . Patients in stage I_EA_ were treated with radiation therapy alone, with radiation doses between 25 and 40 Gy, and patients with stage II_EA_ received systemic chemotherapy with bendamustin/rituximab. Those two patients diagnosed with diffuse large B-cell lymphoma and mantle cell lymphoma received systemic chemotherapy according to the R-CHOP protocol.

**Conclusions:**

Owing to unspecific clinical symptoms, some diagnostic delay may occur in orbital lymphoma. If unspecific orbital symptoms are present, adequate imaging studies followed by early surgical biopsy will contribute to early diagnosis. Once diagnosis is established and staging is complete, radiation therapy is the recommended treatment for stage I_EA_ patients. Systemic chemotherapy is indicated in selected stage II_EA_ patients and in patients with stage III_EA_ disease.

## Background

Orbital lymphomas are rare, comprising only 1% of all non-Hodgkin’s lymphoma [1]. However, lymphomas are the most common primary orbital tumor in adults 60 years of age and older [2]. Margo and Mulla reported in their study of more than 300 orbital malignancies a 55% rate of lymphomas involving the orbit [3]. The majority of non-Hodgkin’s lymphomas of the orbit and orbital adnexa are extranodal marginal-zone B-cell lymphomas of mucosa-associated lymphoid tissue (MALT)-type lymphomas [4].

Although initially described in the stomach and associated with *Helicobacter pylori* infection, MALT lymphomas have subsequently been observed to arise in other epithelial structures, including the thyroid, parotid gland, lung and breast, as well as in the orbit [5-7].

Our study aimed at exploring the diagnostic and therapeutic approach as well as the clinical course of lymphomas involving the orbit.

## Methods

A retrospective review of orbital tumors (*n* = 135) treated at the Department of Cranio-Maxillofacial Surgery from January 2005 to April 2012 retrieved 11 cases of non-Hodgkin’s lymphomas that presented clinically in the orbital region. All 11 patients had been referred to our institution from the Department of Ophthalmology for further diagnosis of an indolent swelling in the orbital/periorbital region.

The initial clinical staging included a computed tomography or magnetic resonance imaging scan of the orbital/periorbital region to localize the tumor site and extension. Based on imaging findings, a surgical biopsy under general anesthesia was planned; in selected patients, depending on the anatomic location, the biopsy was taken using a computer-assisted navigation platform (iPlan CMF 3.0; Brainlab, Feldkirchen, Germany). Histologic diagnosis was initially performed on H & E-stained paraffin sections and additional immunohistochemical staining performed for further immunologic phenotyping. Once diagnosis was confirmed patients were staged according to the Ann Arbor classification. Following complete clinical staging based on the clinical practice of the treating physician, patients were sent to the radiation oncologist or the oncologist for further treatment planning. Post-treatment follow-up visits were organized by the treating physicians. For the purpose of this analysis, the treating physicians were contacted to obtain the latest follow-up information of all patients.

## Results

### Clinical and pathologic features

Eleven patients were included in this analysis. Of the 11 patients, seven were women (64%) and four were men (36%), with a median age of 57.7 years (range, 42.5 to 88.7 years). The most common presenting signs and symptoms were periorbital tumor mass in six patients (55%), exophthalmos in five patients (45%), ocular pain in four patients (36%), and eye motility and visual restrictions in one patient (9%). One patient (9%) was found to have bilateral disease. The tumor was located in the supraorbital region in six patients (55%), the retrobulbar orbital tissue in two patients (18%), and the infraorbital region in three patients (27%). Table 1 displays patient characteristics, including age, gender, site, diagnostic imaging, and surgical treatment.

**Table 1:** **Patient and treatment characteristics of orbital lymphomas (*n*= 11)**

**Characteristic**	**Number of patients**	**%**
Age (years)		
Median	57.7	
Range	42.5 to 88.7	
Gender		
Male	4	36
Female	7	64
Clinical symptoms		
Periorbital swelling	6	55
Proptosis	5	46
Pain	4	36
Epiphora	2	18
Motility impairment	1	9
Ann Arbor stage		
IA	7	64
IIA	2	18
IIIA	2	18
Orbital imaging study		
Computed tomography	10	91
Magnetic resonance imaging	8	73
Staging procedure		
Chest X-ray	5	46
Chest computed tomography	8	73
Abdominal computed tomography	7	64
Bone marrow biopsy	7	64
Surgical treatment		
Surgical biopsy	10	91
Complete surgical removal	1	9
Surgical approach		
Transconjunctival	3	27
Blepharoplasty incision	7	64
Coronal/lateral orbitotomy	1	9
Treatment		
Radiation	7	64
Chemotherapy	4	36

Orbital computed tomography and/or magnetic resonance imaging scans were used in all patients to evaluate the extent of the disease and to aid in radiation treatment planning.

**Figure 1:**
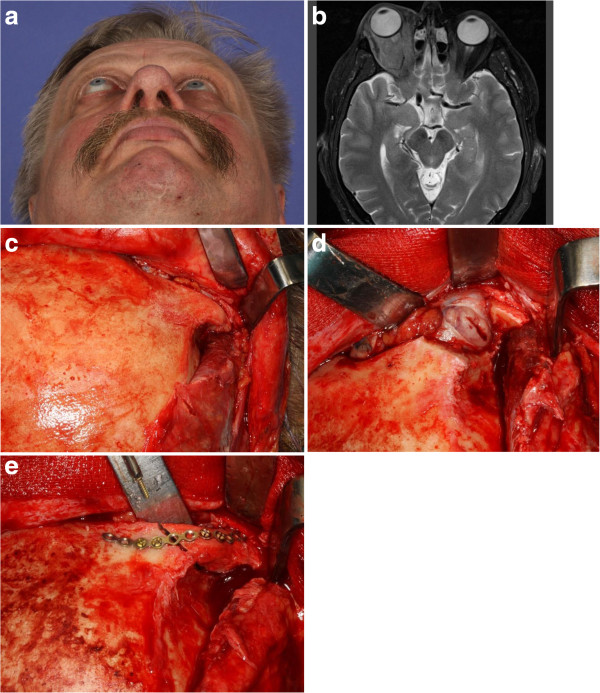
**A patient underwent hemicoronal incision and a lateral orbitotomy approach, followed by a biopsy. (a)** Clinical picture of a 56-year-old patient with a history of right orbital proptosis for 2 months with no visual impairment. **(b)** Axial magnetic resonance imaging scan showing tumor lesion of the intraconal/extraconal compartment of the right orbit. As a diagnostic procedure, a surgical biopsy was performed using a hemicoronal approach: **(c)** the lateral orbital rim was exposed via a hemicoronal approach; **(d)** following temporary osteotomy of the lateral orbital rim, the tumor was exposed and a biopsy was taken; and **(e)** correct anatomic reposition of the right lateral orbital rim and fixation using a 1.3 mm titanium plate.

**Figure 2:**
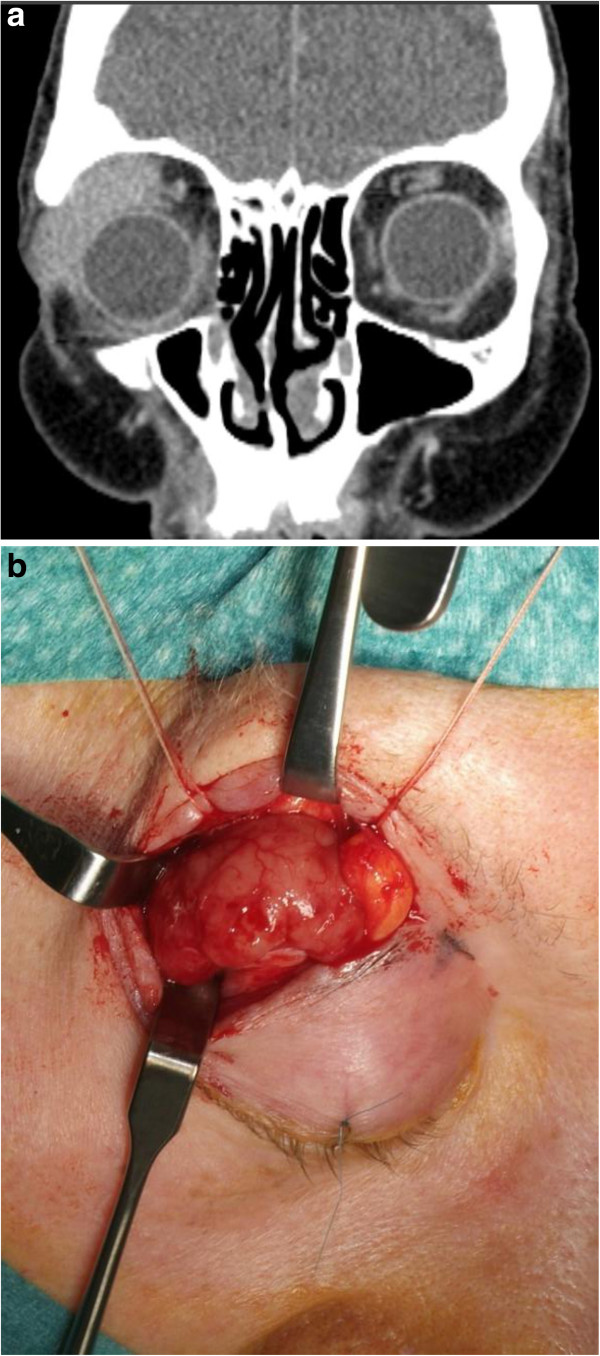
**A patient with involvement of the lacrimal gland received complete surgical removal as primary treatment. (a)** Coronal computed tomography scan showing a tumor lesion in the upper right orbit of a 77-year-old woman. **(b)** The tumor was exposed using a blepharoplasty approach and completely removed together with the lacrimal gland.

Initial treatment consisted of a surgical biopsy under general anesthesia in all patients using various surgical approaches. A blepharoplasty incision was used in seven patients (64%), combined with navigation-assisted biopsy for retrobulbar space location in three patients (27%). A transconjunctival approach was used in three patients (27%). In one patient a biopsy was taken following hemicoronal incision and a lateral orbitotomy approach (Figure 1a to e). One patient with involvement of the lacrimal gland received complete surgical removal as the primary treatment (Figure 2a,b).

Histopathological examination together with immunohistochemical studies were performed on paraffin sections from biopsy specimens of all 11 patients. Extranodal MALT lymphoma was diagnosed in nine patients (82%), and the other two patients were diagnosed with diffuse large B-cell lymphoma and with recurrence of mantle cell lymphoma, respectively.

### Clinical course

Once the diagnosis was confirmed by the pathologist, patients were discussed within the multidisciplinary tumor board and sent for consultation to the oncologist and/or radiation oncologist. Additional clinical staging was performed based on the clinical practice of the treating physician. Staging procedures included chest radiographs or thoracic computed tomography scan, as well as abdominal computed tomography scan. In selected patients a bone marrow biopsy was taken. After complete staging evaluation, seven patients (64%) presented in stage I_EA_, and four patients in stage II_EA_ (*n* = 2) and stage III_EA_ (*n* = 2). Patients with stage I_EA_ MALT lymphoma (*n* = 7) also received radiotherapy as the initial treatment, with radiation given 5 days per week using a conventional fractionation size of 180 to 250 cGy. The mean radiation dose was 3,400 cGy (range, 2,500 to 4,000 cGy). Extraorbital recurrence was detected in only one patient with initial stage I_EA_ after 24 months; this tumor was controlled with salvage radiotherapy. Two patients with stage II_EA_ MALT lymphoma received chemotherapy with bendamustin and rituximab as initial treatment and achieved complete response after four and six cycles of chemotherapy, respectively.

The two patients with mantle cell lymphoma (stage III_EA_) and diffuse large B-cell lymphoma (stage III_EA_) received chemotherapy with R-CHOP (rituximab, cyclophosphamide, doxorubicin,vincristine, and prednisolone) as initial treatment. Both patients achieved complete response after six cycles of R-CHOP.

As of June 2012, information on the survival status was obtained for all 11 patients, with a median follow-up of 0.9 years (range, 0.2 to 6.2 years). Ten patients (91%) are alive with no evidence of disease. One patient with stage I_EA_ MALT lymphoma died 1 year after completion of radiotherapy due to intercurrent disease.

## Discussion

No specific guidelines currently exist for the management of MALT lymphomas, except for localized primary gastric MALT lymphoma. Numerous reports confirm the efficacy of conventional treatment strategies such as surgery, radiotherapy or chemotherapy, alone or in combination, with no significant survival difference [6,8,9]. Surgery as the only treatment modality should not be administered, because there is obviously a high likelihood of local relapse after surgery according to previous reports [10]. The difficulty of complete resection with preservation of function in the orbit may explain the high relapse rate.

Radiation therapy as the initial treatment has been reported to be very effective in MALT lymphoma of the orbit [11-28]. Radiotherapy with a dose range of 25 to 35 Gy seems to be a standard approach because it provides local control and cure for localized orbital lymphoma [11,13,28]. Our data support these findings that excellent local control as well as survival can be achieved in stage I_EA_ MALT lymphoma of the orbit by radiotherapy alone with a mean dose of 34 Gy. However, even after lower radiation dose recommendations in recent publications, there is still some controversy regarding the optimal radiation dose for this tumor [17-19]. Only one extraorbital relapse (one out of nine MALT lymphomas, 11%) with skin infiltration in the upper neck developed 2 years after primary radiotherapy with 36 Gy. Radiation doses above 35 Gy resulted in significant late complications such as cataract formation or keratitis [28]. It should be noted that lens shielding has to be added after a dose of 20 Gy [19]. Interestingly several studies have reported a relatively high local or distant failure rate after successful initial radiotherapy. Jenkins and colleagues reported that 47% of their study population of 192 patients with orbital MALT lymphoma developed extraorbital recurrence after 5 years [29]. Hasegawa and colleagues reported a recurrence rate of 25% out of 20 patients who were treated with radiotherapy alone for orbital MALT lymphoma and developed a relapse in the nonirradiated orbit or at distant sites after a median follow-up of 71 months [14]. In view of some conflicting reports it is difficult to draw final conclusions, but all those sometimes contradictory results point to some variance in the biologic behavior of MALT lymphomas.

There is some evidence that combination chemotherapy is effective in orbital lymphoma [30]. Chemotherapy has never been systematically evaluated in orbital MALT lymphoma because of excellent local control rates in stage IE disease that showed excellent response after primary radiotherapy. Most often, chemotherapy was administered after either surgery or radiotherapy or was reserved for patients with advanced disease stages III_EA_ and IV_EA_. With some ongoing controversial discussion as far as the role of chemotherapy in MALT lymphoma is concerned, it may be concluded that the development of novel systemic strategies particularly for stages III_EA_ and IV_EA_ is needed [8,31]. The clinical efficacy of rituximab, a chimeric mAb directed against the B-cell-specific antigen CD20, was first demonstrated in follicular lymphomas, but the use of the antibody has been extended over the last few years to other subtypes of non-Hodgkin lymphomas with promising results, as a single agent or in combination with chemotherapy [32]. Since CD20 antigen is expressed on the surface of neoplastic cells in virtually all MALT lymphomas, one can assume that rituximab is active in this neoplastic disease. Conconi and colleagues could demonstrate clinical activity in extranodal MALT lymphomas in a phase II study in previously untreated and treated adult patients with a response rate of 73% and a median response duration of 10.5 months [32]. A recent pilot study assessed the tolerability and activity of intralesional injection rituximab in a small group of five patients with orbital B-cell lymphoma, and demonstrated complete remission in two patients and stable disease in two patients [33]. Further clinical trials will show some potential synergistic action between rituximab and chemotherapy and will contribute to better define the role of chemotherapy in extranodal MALT lymphomas.

## Conclusion

MALT lymphomas constitute the majority of orbital and periorbital non-Hodgkin’s lymphomas. Clinical signs and symptoms are unspecific, but a slowly growing painless orbital or periorbital swelling was masking a lymphoma in the majority of our patients. From this perspective, the cranio-maxillofacial surgeon plays an integral part in the multidisciplinary management of patients with orbital lymphomas. Early surgical biopsy together with adequate imaging studies is essential for early and adequate diagnosis of orbital lymphoma. As demonstrated in our patients, radiotherapy is an effective treatment option in patients with stage I_EA_ disease resulting in excellent local tumor control and survival. The role of systemic chemotherapy in MALT lymphoma is still not well defined.

## Consent

Written informed consent was obtained from the patient for publication of this report and any accompanying images.

## Abbreviations

H & E: Hematoxylin and eosin; mAb: Monoclonal antibody; MALT: Mucosa-associated lymphoid tissue; R-CHOP: Rituximab cyclophosphamide, doxorubicin,vincristine, and prednisolone.

## Competing interests

The authors declare that they have no competing interests.

## Authors’ contributions

AME made substantial contributions to conception and design of the manuscript as well as data acquisition. JL and MR have been involved in drafting the manuscript. N-CG was involved in revising the manuscript. All authors read and approved the final manuscript.
